# Enzymatic hydrolyzing performance of *Acremonium cellulolyticus *and *Trichoderma reesei *against three lignocellulosic materials

**DOI:** 10.1186/1754-6834-2-24

**Published:** 2009-10-01

**Authors:** Tatsuya Fujii, Xu Fang, Hiroyuki Inoue, Katsuji Murakami, Shigeki Sawayama

**Affiliations:** 1 Biomass Technology Research Center, National Institute of Advanced Industrial Science and Technology (AIST), Hiroshima 737-0197, Japan

## Abstract

**Background:**

Bioethanol isolated from lignocellulosic biomass represents one of the most promising renewable and carbon neutral alternative liquid fuel sources. Enzymatic saccharification using cellulase has proven to be a useful method in the production of bioethanol. The filamentous fungi *Acremonium cellulolyticus *and *Trichoderma reesei *are known to be potential cellulase producers. In this study, we aimed to reveal the advantages and disadvantages of the cellulase enzymes derived from these fungi.

**Results:**

We compared *A. cellulolyticus *and *T. reesei *cellulase activity against the three lignocellulosic materials: eucalyptus, Douglas fir and rice straw. Saccharification analysis using the supernatant from each culture demonstrated that the enzyme mixture derived from *A. cellulolyticus *exhibited 2-fold and 16-fold increases in Filter Paper enzyme and β-glucosidase specific activities, respectively, compared with that derived from *T. reesei*. In addition, culture supernatant from *A. cellulolyticus *produced glucose more rapidly from the lignocellulosic materials. Meanwhile, culture supernatant derived from *T. reesei *exhibited a 2-fold higher xylan-hydrolyzing activity and produced more xylose from eucalyptus (72% yield) and rice straw (43% yield). Although the commercial enzymes Acremonium cellulase (derived from *A. cellulolyticus*, Meiji Seika Co.) demonstrated a slightly lower cellulase specific activity than Accellerase 1000 (derived from *T. reesei*, Genencor), the glucose yield (over 65%) from lignocellulosic materials by Acremonium cellulase was higher than that of Accellerase 1000 (less than 60%). In addition, the mannan-hydrolyzing activity of Acremonium cellulase was 16-fold higher than that of Accellerase 1000, and the conversion of mannan to mannobiose and mannose by Acremonium cellulase was more efficient.

**Conclusion:**

We investigated the hydrolysis of lignocellulosic materials by cellulase derived from two types of filamentous fungi. We found that glucan-hydrolyzing activity of the culture supernatant from *A. cellulolyticus *was superior to that from *T. reesei*, while the xylan-hydrolyzing activity was superior for the cellulase from *T. reesei*. Moreover, Acremonium cellulase exhibited a greater glucan and mannan-hydrolyzing activity than Accellerase 1000.

## Background

Lignocellulosic biomass represents a promising starting material for the production of bioethanol fuel, as it contains a large quantity of sugars in the form of cellulose and hemicellulose. Ethanol fuel production from lignocellulosic biomass is advantageous as it does not lead to competition for food resources [1]. For ethanol fuel production from lignocellulosic materials, cellulose and hemicellulose must firstly be hydrolyzed to fermentable sugars. Sulfuric acid and cellulolytic enzymes are the major hydrolyzers of cellulose and hemicellulose identified to date [2]. However, when sulfuric acid is used for the hydrolysis of lignocellulosic materials, it is necessary to remove the residual sulfuric acid from the hydrolyzing solution prior to yeast fermentation. Furthermore, sulfuric acid produces toxic compounds that inhibit fermentation [2-4]. Therefore, enzymatic saccharification of lignocellulosic materials that does not require the use of acidic compounds represents an important improvement in the generation of fermentable sugars during the bioethanol production process. The development of efficient pretreatment methods that do not require the use of chemicals before enzymatic hydrolysis, such as milling treatment, have also been eagerly investigated [5,6]. Cellulases, a group of enzymes that hydrolyze crystalline cellulose to smaller oligosaccharides and subsequently glucose, and hemicellulases that hydrolyze hemicellulose to monomeric sugars, have been used for the enzymatic saccharification of lignocellulosic materials.

Filamentous fungal strains, also sometimes termed wood-degrading organisms, secrete a large quantity of cellulase and hemicellulase [7-10]. Cellulase produced by fungi comprises three major enzyme components: 1) endoglucanases that randomly hydrolyze internal glycosidic linkages; 2) cellobiohydrolases that produce cellobiose from cellulose chain ends; and 3) β-glucosidases that convert cellobiose to glucose [10]. Xylan-hydrolyzing enzymes including xylanase and β-xylosidase, and mannan-hydrolyzing enzymes such as mannanase and β-mannosidase are examples of hemicellulases produced by fungi [11]. Of the cellulases and hemicellulases produced by fungi, the enzymes derived from *Trichoderma reesei *represent the best characterized, and are often used for enzymatic saccharification of lignocellulosic materials [12]. The genome of *T. reesei *QM6a has been sequenced and the sequence information is readily available [13]. The cellulase derived from *T. reesei *demonstrates a relatively weak β-glucosidase activity, and the reaction from cellobiose to glucose has been shown to be slow [14]. Yamanobe *et al*. isolated enzymes from the filamentous fungus strain *Acremonium cellulolyticus *Y-94, which produces high levels of cellulase [15]. These enzymes demonstrated a significantly higher β-glucosidase activity than the cellulase derived from *T. reesei*. However, the cellulase and hemicellulase produced by *A. cellulolyticus *have not been as well characterized as those produced by *T. reesei*. A number of cellulase hyperproducing mutants have also been obtained from *A. cellulolyticus *Y-94 and *T. reesei *QM6a following treatment with mutagens including ultraviolet (UV) and various chemical compounds [12,16-18]. Understanding the advantages and disadvantages of each of the enzymes derived from *A. cellulolyticus *and *T. reesei *may prove essential for the improvement of their hydrolyzing performance during bioethanol production processes.

The aim of this study was to further understand cellulases derived from *A. cellulolyticus *and *T. reesei *by analyzing the hydrolysis of lignocellulosic biomass. We determined the specific activity of cellulase and hemicellulase, and performed enzymatic saccharification of three lignocellulosic materials.

## Methods

### Enzymes

The cellulase hyperproducing strains used in this study were *A. cellulolyticus *CF-2612 [16] and *T. reesei *CDU-11 [19]. *A. cellulolyticus *CF-2612 was cultured in production medium in Erlenmeyer flasks [17], and the resulting culture supernatant was analyzed. The culture supernatant from *T. reesei *was kindly supplied by Kyowa Hakko Kougyo Co. (Tokyo, Japan). The commercial enzymes used in this study were Acremonium cellulase (AC, derived from *A. cellulolyticus*; Meiji Seika, Tokyo, Japan) and Accellerase 1000 (derived from *T. reesei*; Genencor, Rochester, NY, USA).

### Enzyme assay

The soluble protein concentration was determined using the method of Lowry *et al*. [20]. Filter-paper (FPase) activity was measured as described previously by Ghose [21], a method recommended by the Commission of Biotechnology, IUPAC. The activity of CMCase was assayed based on the method of Mandels *et al*. [22]. Briefly, appropriately diluted supernatant and 0.5 ml of carboxymethylcellulose (CMC, 2% w/v) in citrate buffer (50 mM, pH 4.8) were mixed in equal volumes, and the enzyme reaction mixture was incubated at 50°C for 30 min. Avicelase activity was determined under similar conditions, with the exception that the enzyme reaction proceeded for 2 h in 1.0 ml of acetate buffer (0.1 M, pH 4.8), 10 mg of Avicel PH-101 (Fluka, Buchs, Switzerland) as the substrate and 1.0 ml of diluted enzyme solution. The reducing sugars released were analyzed via the dinitrosalicylic acid (DNS) assay [17]. Xylanase activity was assayed in a 1.0-ml reaction mixture containing 1% (w/v) birchwood xylan (Sigma-Aldrich, St. Louis, MO, USA), 50 mM acetate buffer (pH 5.0) and appropriately diluted enzyme solutions. Following 30 min incubation at 45°C, the reducing sugar liberated was measured using the DNS assay. Mannanase activity was measured under similar conditions as xylanase activity, with the exception that 1% glucomannan (Megazyme, Wicklow, Ireland) served as the substrate. For the measurement of β-glucosidase activity, appropriately diluted enzyme solution and 10 mM *p*-nitrophenyl-β-D-glucopyranoside (Wako Pure Chemical Industries, Tokyo, Japan) were added to 100 mM citrate buffer, and the enzyme reaction mixture was incubated at 45°C for 10 min. Absorbance at 420 nm was then measured. β-Xylosidase and β-mannosidase activities were measured under similar conditions, with the exception that *p*-nitrophenyl-β-D-xylopyranoside and *p*-nitrophenyl-β-D-mannopyranoside (Wako Pure Chemical Industries) were used as the substrates, respectively. For these experiments, one unit of enzyme activity was defined as the amount of enzyme required to produce 1 μmol of reducing sugar per minute.

### Pretreatment of lignocellulosic materials

Eucalyptus and Douglas fir wood chips were kindly supplied by a pulp factory located close to our research center (Oji Paper Co., Tokyo, Japan). Rice straw was kindly supplied by Dr Tokuyasu (National Food Research Institute, Tsukuba, Japan). Ball-milling pretreatment of cellulosic materials was based on the methods described by Inoue *et al*. [5]. The initial composition of eucalyptus (40.0% glucan and 10.4% xylan) and Douglas fir (51.9% glucan and 13.2% mannan) was determined as described previously [5]. The initial composition of rice straw (37.0% glucan and 13.7% xylan) was determined by the method of Hideno *et al*. [6].

### Enzymatic hydrolysis

Enzymatic hydrolysis was performed using an enzyme constituting 22.5 or 90.0 mg protein per gram of dry substrate. The soluble protein concentration was determined using the method of Lowry *et al*. [20]. The diluted enzyme solution (8 ml) in 50 mM acetate buffer (pH 5.0) was added to 2 g of pretreated material in a 15-ml tube. The reaction mixture was then incubated at 50°C for 72 h with mixing using a magnetic stirrer. The hydrolysate was centrifuged, and the supernatant analyzed. This experiment was performed in triplicate.

### Analysis of substrate hydrolysates

Substrate hydrolysates were analyzed using a high-performance liquid chromatography (HPLC) system equipped with a RI-2031 Plus detector (Jasco, Tokyo, Japan). Glucose, xylose, mannose and mannobiose were analyzed using an Aminex HPX-87P column (Bio-Rad, Hercules, CA, USA) fitted with a Carbo-P micro-guard cartridge. The mobile phase used was doubly deionized water, and the flow rate was 1.0 ml/min at a column temperature of 80°C.

## Results

### Saccharification analysis of the culture supernatant

In order to investigate the features of cellulase and hemicellulase derived from *A. cellulolyticus *and *T. reesei*, we first measured FPase, Avicelase, CMCase and β-glucosidase specific activity to determine cellulase activity, and xylanase, β-xylosidase, mannanase and β-mannosidase specific activity to determine hemicellulase activity (Table 1). The enzyme mixtures that were used to analyze these parameters were isolated from the culture supernatant produced by *A. cellulolyticus *CF-2612 (SCF-2612) and *T. reesei *CDU-11 (SCDU-11). We found that SCF-2612 contained higher FPase, Avicelase, CMCase and β-glucosidase specific activity than SCDU-11. In particular, β-glucosidase activity in SCF-2612 demonstrated a greater than 16-fold increase in activity compared with that in SCDU-11. In contrast, xylanase, β-xylosidase and β-mannosidase specific activities for SCDU-11 were higher than those observed for SCF-2612. Mannanase specific activity remained similar for the two enzymes.

**Table 1:** Specific activities of cellulases and hemicellulases derived from *A. cellulolyticus *and *T. reesei*.

**Enzyme**		**Specific activity (U mg^-1 ^protein)**							
		
		**FPase**	**Avicelase**	**CMCase**	**β-glucosidase**	**Xylanase**	**β-xylosidase**	**Mannanase**	**β-mannosidase**
culture supernatant	SCF-2612	0.66 ± 0.13	0.26 ± 0.02	4.52 ± 1.32	1.20 ± 0.02	12.4 ± 0.15	0.011 ± 0.001	1.10 ± 0.15	0.00045 ± 0.0001
	SCDU-11	0.25 ± 0.05	0.11 ± 0.03	3.55 ± 1.01	0.072 ± 0.003	25.4 ± 2.32	0.049 ± 0.001	1.20 ± 0.06	0.0078 ± 0.0004
commercial enzymes	Accellerase 1000	0.44 ± 0.01	0.25 ± 0.01	6.75 ± 0.34	2.85 ± 0.06	8.86 ± 0.85	0.023 ± 0.001	0.29 ± 0.03	0.00084 ± 0.0001
	Acremonium cellulase	0.41 ± 0.02	0.19 ± 0.05	4.44 ± 0.87	2.09 ± 0.24	6.21 ± 0.21	0.0074 ± 0.0001	4.88 ± 0.87	0.051 ± 0.001

Data represents the mean of three experiments.

**Figure 1:**
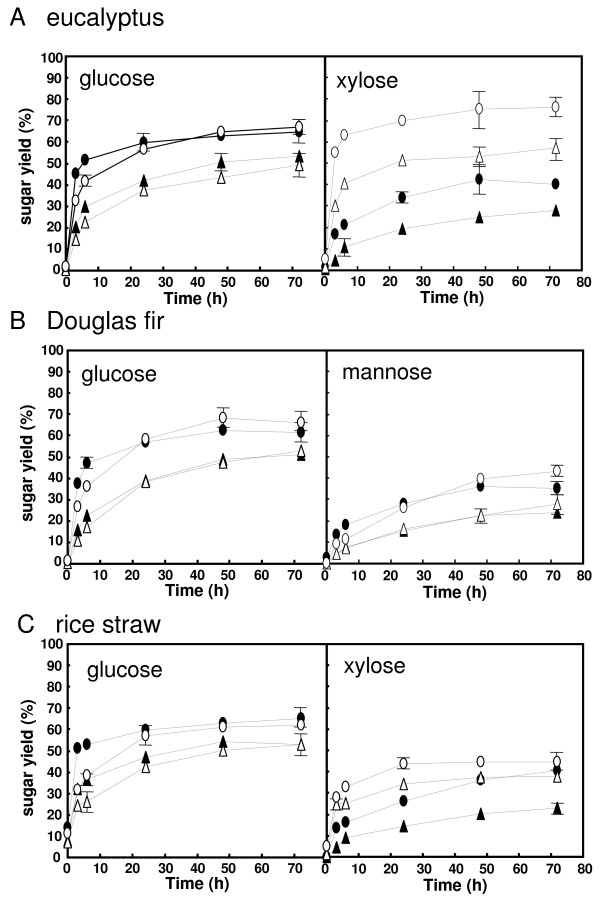
**Saccharification of pretreated lignocellulosic materials using culture supernatants**. Sugar production from eucalyptus (A), Douglas fir (B) and rice straw (C) by SCF-2612 (filled) and SCDU-11 (open). Enzyme content used in the assay was 90.0 mg protein g^-1 ^substrate (circles) and 22.5 mg protein g^-1 ^substrate (triangles). Data are presented as the mean of three individual experiments. Standard deviation of a point without error bar is under the 3%.

We next examined the saccharification ability of these enzymes during time-course hydrolysis of three ball-milled lignocellulosic materials, eucalyptus, Douglas fir and rice straw. We found that SCDU-11 and SCF-2612 produced an equivalent amount of glucose from each material at 72 h. However, the SCF-2612 (90 mg protein g^-1 ^substrate) glucose yield at 3 h for eucalyptus, Douglas fir and rice straw was 47%, 38% and 51%, respectively, while the SCDU-11 glucose yield was 32%, 27% and 32%, respectively (Figure 1). SCF-2612 also produced a greater amount of glucose at 3 h than SCDU-11 when we used 22.5 mg protein g^-1 ^substrate for the enzymes (Figure 1). This data suggests that the conversion of cellulose to glucose by SCF-2612 was faster than that of SCDU-11, which could be caused by a higher cellulase activity of SCF-2612 (Table 1).

Eucalyptus and rice straw contain hemicellulose, mainly in the form of xylan, while Douglas fir mainly contains mannan [5,6]. Thus, we examined xylose production from eucalyptus and rice straw, and mannose production from Douglas fir for each of the culture supernatants. The maximum xylose yield obtained from eucalyptus was 72% and from rice straw was 43% for SCDU-11 (Figure 1A and 1C). This data is consistent with that of the xylanase and β-xylosidase activities, which were higher for SCDU-11 than for SCF-2612 (Table 1). Mannose production from Douglas fir was similar between the two supernatants (Figure 1B). The HPLC column used in this study was not able to completely separate mannose and mannobiose, so the mannose production value presented also includes mannobiose. Mannanase specific activities of SCDU-11 and SCF-2612 were similar; however, β-mannosidase specific activity of SCDU-11 was 17-fold higher than that of SCF-2612 (Table 1). Thus, the SCF-2612 hydrolysate may contain more mannobiose than that of SCDU-11.

### Saccharification analysis of commercial enzymes

We investigated the cellulase and hemicellulase specific activity of AC derived from *A. cellulolyticus *and of Accellerase 1000 derived from *T. reesei*. We revealed that the FPase, Avicelase, CMCase, β-glucosidase, xylanase and β-xylosidase specific activities of Accellerase 1000 were slightly higher than those achieved by AC (Table 1). In contrast, AC contained higher mannanase and β-mannosidase specific activities than Accellerase 1000 (Table 1).

**Figure 2:**
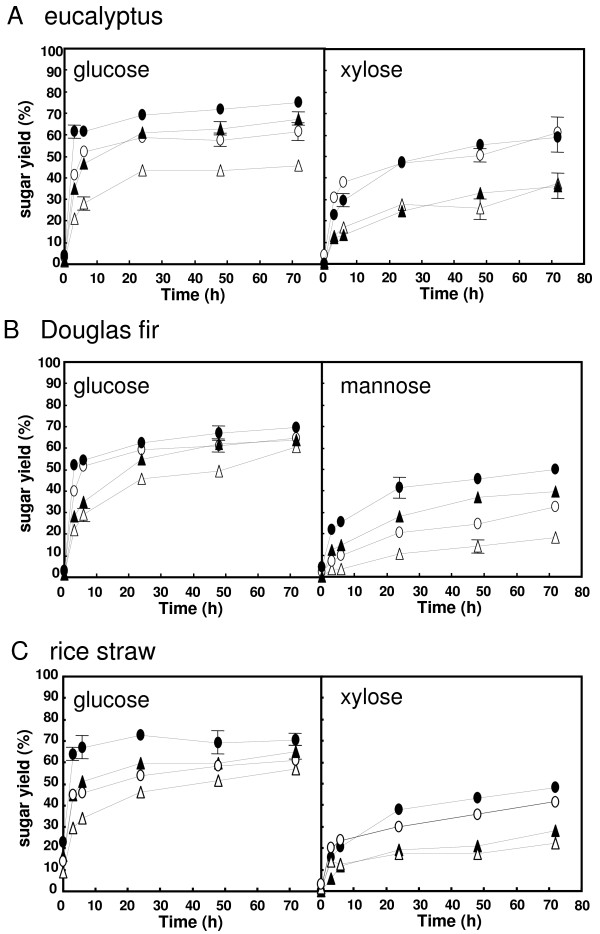
**Saccharification of pretreated lignocellulosic materials using commercially available enzymes**. Sugar production from eucalyptus (A), Douglas fir (B) and rice straw (C) by Acremonium cellulase (filled) and Accellerase 1000 (open). Enzyme content used in the assay was 90.0 mg protein g^-1 ^substrate (circles) and 22.5 mg protein g^-1 ^substrate (triangles). Data are presented as the mean of three individual experiments. Standard deviation of a point without error bar is under the 3%.

**Figure 3:**
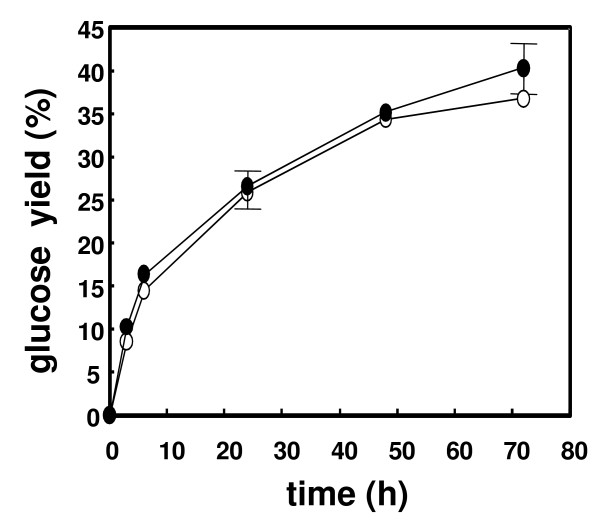
**Saccharification of Avicel by commercial enzymes**. Time-course of glucose production by Acremonium cellulase (filled) and Accellerase 1000 (open). Enzyme content used in the assay was 22.5 mg protein g^-1 ^substrate. Data are means of three experiments. Glucose yield was calculated by assuming that Avicel contains 100% glucan. Standard deviation of a point without error bar is under the 3%.

We next examined saccharification of these commercial enzymes during hydrolysis of three ball-milled lignocellulosic materials in a time-dependent manner. Although the cellulase activity of AC was found to be slightly lower than that of Accellerase 1000 (Table 1), the maximum glucose yield from each material that AC produced (greater than 69% yield) was larger than that of Accellerase 1000 (less than 60% yield) (Figure 2). Furthermore, the amount of glucose produced by AC at 3 h for eucalyptus (62%), Douglas fir (52%) and rice straw (65%) was greater than that produced by Accellerase 1000 (eucalyptus, 42%; Douglas fir, 40%; rice straw, 45%). These results demonstrate the faster conversion of cellulose to glucose by AC. The hydrolysis curves for Avicel were found to be similar for both Accellerase 1000 and AC (Figure 3), suggesting that the differences in hydrolysis performance between these enzymes were dependent on the substrate (see discussion).

The xylose yields from eucalyptus and rice straw were found to be similar for both Accellerase 1000 and AC (Figure 2A and 2C), although the xylanase and β-xylosidase activities of AC were lower than those of Accellerase 1000. In contrast, the maximum mannose yield obtained from Douglas fir by AC was 49% (Figure 2B). This result is consistent with the higher mannanase and β-mannosidase activity levels obtained by AC compared with those of Accellerase 1000.

## Discussion

*T. reesei *generally produce lower levels of β-glucosidase activity than *A. cellulolyticus*. This study demonstrated that the β-glucosidase specific activity of SCDU-11 was significantly lower than that of SCF-2612 (Table 1). This lower β-glucosidase activity is thought to trigger accumulation of cellobiose, which is a strong inhibitor of cellobiohydrolase and endoglucanase activities during cellulose hydrolysis [9]. In fact, our data demonstrated that SCDU-11 hydrolyzed lignocellulosic materials more slowly than SCF-2612.

Analysis of saccharification of eucalyptus, Douglas fir and rice straw demonstrated that AC was able to produce more glucose than Accellerase 1000, even though it exhibited a lower cellulase specific activity. Similar results were also obtained during the analysis of xylose production. A potential explanation of this result may be the differences in substrate composition. The substrate used for the measurement of cellulase activity was highly purified and contained little material other than cellulose. However, lignocellulosic materials contain additional elements including lignin, which is known to inhibit the cellulose-hydrolyzing reaction [23]. The hydrolysis curves of Avicel, that were pure for cellulose, were similar for both Accellerase 1000 and AC (Figure 3). This data suggest that Accellerase 1000 may be more sensitive to cellulase inhibitors, such as lignin, than AC. Enzyme sensitivity to inhibitors may aid in the explanation as to why xylose yield was equivalent for these enzymes. However, we do not have a clear explanation for this phenomenon.

It is well established that cellulose-degrading fungi produce cellulase and hemicellulase complexes [7-9,11]. In this study, the culture supernatant derived from *A. cellulolyticus *was found to exhibit a lower xylan-hydrolyzing activity than that derived from *T. reesei*. *A. cellulolyticus *CF-2612 has been screened previously as a high cellulase producer, rather than a high hemicellulase producer, in *A. cellulolyticus *Y-94 mutagenesis studies involving UV irradiation and chemical compounds [16,17]. *A. cellulolyticus *Y-94 demonstrated a higher xylan-hydrolyzing activity than *A. cellulolyticus *CF-2612 (unpublished data), suggesting that the genes related to xylan-hydrolyzing performance of *A. cellulolyticus *CF-2612 may have been mutated, thus resulting in a reduction in xylan-hydrolyzing activity. The reduced xylan-hydrolyzing activity of *A. cellulolyticus *CF-2612 may also prove problematic for the saccharification of lignocellulosic materials and may require improvement. The maximum mannose yield produced from Douglas fir was less than 50% in all experiments in this study. This finding indicates that the mannan-hydrolyzing activity of *A. cellulolyticus *and *T. reesei *strains used was insufficient. We are currently focusing on producing an *A. cellulolyticus *strain that exhibits a higher mannan-hydrolyzing activity.

In this study, AC generated glucose from lignocellulosic materials more efficiently than SCF-2612, while the glucose production curves were similar for both Accellerase 1000 and SCF-2612 (Figures 1 and 2). We also found that the FPase and Avicelase specific activities of AC and Accellerase 1000 were lower than those of SCF-2612 (Table 1). However, the β-glucosidase specific activity of AC and Accellerase 1000 was 2.4 and 1.7-fold higher than that of SCF-2612, respectively. The ratio of β-glucosidase/FPase activity for SCF-2612 (1.8) was also lower than that for AC (5.1) and Accellerase 1000 (6.5). These results suggest that β-glucosidase is important for hydrolyzing lignocellulosic materials. The improvement of β-glucosidase activity in fungal strains involved in the hydrolysis of lignocellulosic materials requires further study. However, given that commercial enzymes often contain additional compounds including enzyme stabilizers, it may prove difficult to directly compare the activities of these enzymes with those present in culture supernatants.

## Conclusion

In this study, we investigated the cellulase and hemicellulase specific activities of both culture supernatants and commercial enzymes derived from *A. cellulolyticus *and *T. reesei*, and examined their performance during the saccharification of three lignocellulosic materials. The culture supernatant derived from *A. cellulolyticus *demonstrated a higher cellulase specific activity and glucose yield from lignocellulosic materials than the *T. reesei *supernatant. In contrast, the enzymes derived from *T. reesei *demonstrated a superior xylan-hydrolyzing activity than those derived from *A. cellulolyticus*. AC produced a greater amount of glucose from lignocellulosic materials and a higher mannan-hydrolyzing activity than Accellerase 1000. Further studies will aid in the development of cellulases and hemicellulases that hydrolyze lignocellulosic materials more efficiently during the bioethanol production process, for advancing the generation of alternative fuel sources.

## Competing interests

The authors declare that they have no competing interests.

## Authors' contributions

TF carried out the measurement of cellulase and hemicellulase activities in SCF-2612 and SCDU-11, the enzymatic hydrolysis and drafted the manuscript. XF and HI carried out the measurement of cellulase and hemicellulase activities of the commercial enzymes. XF, HI, KM and SS designed and coordinated the study and helped in the drafting of the manuscript. All authors read and approved the final manuscript.
